# Overexpression of MicroRNA-10a in Germ Cells Causes Male Infertility by Targeting Rad51 in Mouse and Human

**DOI:** 10.3389/fphys.2019.00765

**Published:** 2019-06-18

**Authors:** Huihui Gao, Hui Wen, Congcong Cao, Daqian Dong, Chenhao Yang, Shengsong Xie, Jin Zhang, Xunbin Huang, Xingxu Huang, Shuiqiao Yuan, Wuzi Dong

**Affiliations:** ^1^College of Animal Science and Technology, Northwest A&F University, Yangling, China; ^2^Institute of Reproductive Health, Tongji Medical College, Huazhong University of Science and Technology, Wuhan, China; ^3^Key Laboratory of Agricultural Animal Genetics, Breeding, and Reproduction, Ministry of Education and Key Laboratory of Swine Genetics and Breeding, Ministry of Agriculture, Huazhong Agricultural University, Wuhan, China; ^4^Wuhan Tongji Reproductive Medicine Hospital, Wuhan, China; ^5^School of Life Sciences and Technology, ShanghaiTech University, Shanghai, China; ^6^Shenzhen Huazhong University of Science and Technology Research Institute, Shenzhen, China

**Keywords:** miRNA-10a, spermatogenesis, overexpression mice, Rad51, meiosis, non-obstructive azoospermia

## Abstract

Spermatogenesis is a complicated process including spermatogonial stem cells self-renewal and differentiates into mature spermatozoa. MicroRNAs (miRNAs) as a class of small non-coding RNAs play a crucial role during the process of spermatogenesis. However, the function of a plenty of miRNAs on spermatogenesis and the potential mechanisms remain largely unknown. Here, we show that genetically conditional overexpressed miR-10a in germ cells caused complete male sterility, characterized by meiotic arrested in germ cells. Analysis of miR-10a overexpression mouse testes reveals that failure of double strand break (DSB) repairs and aberrant spermatogonial differentiation. Furthermore, we identified *Rad51* as a key target of miR-10a in germ cell by bioinformatics prediction and luciferase assay, which may be responsible for the infertility of the miR-10a overexpressed mice and germ cell arrested patients. Our data show that miR-10a dependent genetic regulation of meiotic process is crucial for male germ cell development and spermatogenesis in both mouse and human. These findings facilitate our understanding of the roles of miRNA-10a in spermatogenesis and male fertility.

## Introduction

Mammalian spermatogenesis is a complex cellular differentiation process through which male germline stem cells develop sequentially into spermatogonia, spermatocytes, spermatids, and eventually spermatozoa. This process can be divided into three phases based upon the major cellular events occurring, which called mitosis (spermatogonial proliferation and differentiation), meiosis (reduction of chromosomal number from diploid to haploid), and spermiogenesis (spermatid differentiation into spermatozoa; de Kretser et al., 1998). These cellular events require precise spatiotemporal expression of specific protein-coding genes, which are tightly controlled at both the transcriptional and post-transcriptional levels. In particularly, meiosis is the key developmental program of gametogenesis during which haploid gametes are generated to cope with the doubling chromosome number that occurs after fertilization. During spermatogenesis, large number of mRNA translation is uncoupled with their transcription due to an earlier transcriptional cessation in post-meiotic stage (Idler and Yan, 2012). Translational repression of mRNAs that are transcribed in spermatocytes and/or early spermatids must be achieved through a post-transcriptional regulatory mechanism, which still remains elusive.

MicroRNAs (miRNAs) are a family of short (18–23 nucleotides), single stranded, non-coding RNA molecules that regulate post-transcriptional gene silencing in many organisms by binding to specific base pairs on their target mRNAs, thereby inducing translational repression (Gregory et al., 2005; Comazzetto et al., 2014). Recently, several studies have demonstrated that miRNA dysregulation has been implicated in male fertility lead to sperm abnormality and spermatogenesis disruption (Tian et al., 2013; Yao et al., 2015; Yuan et al., 2015b), suggesting that miRNA are functionally important in the process of spermatogenesis. For instance, inhibition miR-188-3p led to elevation of apoptotic level of spermatogenic cells by regulating its target gene, *Mlh1*, an essential gene for homologous chromosome pairing during meiosis (Song et al., 2017), and miR-221, miR-203, and miR-34b were reported to regulate meiotic progression by targeting *c-Kit* and *Cdk6*, coordinately (Smorag et al., 2012). Additionally, miR-34 family (miR-34b/c) was abundantly expressed in mammalian gametes and has been demonstrated to play an essential role in spermatogenesis by regulation of post-transcription (Bouhallier et al., 2010; Bao et al., 2012; Comazzetto et al., 2014; Tscherner et al., 2014; Yuan et al., 2015b). Nearly 1000 miRNAs have been identified in the mouse and human genomes, and it is very likely that other many miRNAs also regulate spermatogenesis. However, the function and mechanisms of individual miRNA in regulating mammalian germ cells development and meiosis are largely unknown and research on this topic is still in its infancy.

miRNA-10a (miR-10a) was firstly reported to be highly expressed in Thy^+^ SSC-enriched testis cell population via small RNA-Seq, which suggesting that miR-10a play a critical role in spermatogenesis (Niu et al., 2011). miR-10a has also been reported to contribute to retinoic acid induced smooth muscle cell differentiation and its expression levels can be regulated by retinoic acid (Weiss et al., 2009; Huang et al., 2010; Khan et al., 2015). Importantly, all-trans retinoic acid and its nuclear receptors are the major players of gametogenesis and are instrumental to fertility in both sexes (Teletin et al., 2017). Therefore, all of those evidences are indirectly suggested that miR-10a may be a major player in the processes of spermatogenesis and male fertility. Interestingly, loss function of miR-10a in mice did not display observed pathologies, except exhibit increased frequency with of high-grade dysplasia and higher incidence of tubule-villous adenomas in female Apc (min) heterozygote background mouse model (Stadthagen et al., 2013). However, there are lack of any direct information to decipher the role and mechanism underlying of miR-10a in spermatogenesis and male fertility to date. Thus, in this study, we generated a germ cells specific conditional overexpressed miR-10a mouse model through Cre/loxp system to determine the physiological role and underline mechanism of miR-10a during mammalian spermatogenesis. Our aim was to extend a broad view of individual miRNA functions in spermatogenesis through genetic approaches and to determine the molecular mechanism of miR-10a control germ cell development in both human and mice. Our data provides a useful resource to further understand the regulatory role of miR-10a in spermatogenesis.

## Materials and Methods

### Ethics Statement

Ethical approval for this study was provided by the Ethics Committee of Tongji Medical College, Huazhong University of Science and Technology, and written informed consent was obtained from all patients before sample collection.

All animal procedures were approved by the Institutional Animal Care and Use Committee of Northwest A&F University in China, and the mice were housed in the specific pathogen free facility of Northwest A&F University.

### Human Samples

All human testicular samples in this study were abandoned biopsy testicular tissue after treatment, and collected according to protocols approved by the Medical Ethics Committee from the Center for Reproductive Medicine, Tongji Medical College, Huazhong University of Science and Technology. All patients signed informed consent for the collection and use of their samples for this study. Testicular samples were obtained from non-obstructive azoospermia (NOA), obstructive azoospermia (OA) patients (aged 22–40 years) with who underwent micro-TESE at the Center for Reproductive Medicine, Huazhong University of Science and Technology.

### Generation of Male Germ Cell-Specific Conditional Over-Expression miR-10a Transgenic Mice

A precursor of mouse miR-10a (pri-miR-10a) was amplified from C57BL/6J mouse genomic DNA using standard PCR reactions (Xie et al., 2013) with the following primers: forward: 5′-ATCctcgagAATAATTCATGCGCCACCGA-3′ (Xho I) and reverse: 5′-GTAgcggccgcCACCAACATCACACGCACAAG-3′ (Not I). The 791bp product was double digested with two restriction endonucleases Xho I and Not I, and then cloned into the modified transgenic expression vector pUBC (CMV-LoxP-GFP-STOP-LoxP), which was found to have reliable expression across different cell types (Xie et al., 2013). The modified vector CMV-LoxP-GFP-STOP-LoxP-mir10a containing a synthetic rTA-polyA-TetO-CMV element, pri-miR-10a was ligated after LoxP-GFP-STOP-LoxP element. The CMV-LoxP-GFP-STOP-LoxP-mir10a transgenic cassette containing a pri-mir-10a genetic fragment, which was linearized by SspI and SgrDI and injected into fertilized eggs derived from C57BL/6 mice to generate CMV-LoxP-GFP-STOP-LoxP-mir10a transgenic mice. All mouse injections were carried out in Model Animal Research Center of Nanjing University in China. Primers used for the identification of CMV-LoxP-GFP-STOP-LoxP-mir10a transgenic mice are as follows: tg-m10a-forward: 5′-ACATTTCTCTCCGGAACCCTTA-3′ and tg-m10a-reverse: 5′-GCTGGTTCTTTCCGCCTC-3′, the size of PCR product was 437bp. To obtain male germ cell-specific conditional over-expression of miR-10a transgenic mice, female transgenic mice were mated with Ddx4-Cre transgenic male mice. Primers used for the identification of Ddx4-Cre lines are as follows: Cre-forward: 5′-ACCAGGTTCGTTCACTCATG-3′ and Cre-reverse: 5′-CTTAGCGCCGTAAATCAATC-3′, the size of PCR product was 401bp.

### Cell Culture

HEK293T, GC-1 and NIH3T3 cell lines were cultivated in Dulbecco’s modified Eagle’s medium (DMEM; Hyclone, United States) supplemented with 10% FBS (Gibco, United States) and 1% penicillin–streptomycin (Hyclone, United States) at 37°C containing 5% CO_2_ in a humidified incubator. Lipofectamine 2000 (Invitrogen) was used for transfection according to the manufacturer’s instructions.

### Histology and Immunocytochemistry Analyses

Testes from WT and miR-10a overexpressed (*Ddx4-Cre; miR-10a^lox/lox^*, herein called Ddx4-cOE) mice were dissected and fixed in Bouin’s solution overnight at 4°C followed by paraffin embedding. Paraffin sections (5 μm) were cut and stained with the periodic acid-Schiff (PAS) staining or H&E staining for histological analyses after de-paraffinization and rehydration using a series of graded ethanol. TUNEL staining was performed on Paraffin sections by using the *in situ* Cell Death Detection Kit (11684817910, Roche) according to the manufacturer’s instructions.

For immunofluorescence staining of testes, the cryosections were prepared as previously described (Yuan et al., 2015a). Briefly, the slides were permeabilized by 0.5% Triton-X 100 for 15 min at room temperature, washed three times with PBS, for 5 min each time. Heat-mediated antigen retrieval was performed in boiling citrate buffer (pH 6.0) for 10 min, then the slides cooled down to room temperature. After washed with PBS, the sections were treated with blocking buffer (30 μl FBS + 30 μl normal goat serum + 940 μl 1% BSA in PBS) for 1 h at room temperature. Slides were then incubated overnight at 4°C with primary antibodies following appropriate FITC-conjugated secondary antibodies (Invitrogen Molecular Probes). DAPI was used to stain the nucleus and images were obtained using fluorescent microscope (Nikon, Japan). The following primary antibodies were used: DDX4 (1:1000, Cat: ab13840, Abcam), SCYP3 (1:800, Cat: ab15093, Abcam), γ-H2AX (1:500, Cat: ab26350, Abcam), SYCP1 (1:750, Cat: ab15090, Abcam), GATA4 (1:50, Cat: ab84593, Abcam), STRA8 (1:250, Cat:ab 49602, Abcam), PLZF (1:400, Cat: sc-28319, Santa Cruz).

### Meiotic Chromosome Spreads Assays

Chromosome spreads of prophase I spermatocytes were performed as previously described with slightly modified (Kolas et al., 2005). Briefly, testes were collected from WT and Ddx4-cOE mice, de-capsulated into 100 mM sucrose and chopped/pipetted to release germ cells. Cells were added to slides coated with 1% paraformaldehyde and dried in a humidified chamber. Slides were then washed with 0.4% Photo-Flo 200 Solution (Electron Microscopy Sciences), dried well and stored at -80°C. For immunofluorescence, meiotic spreads were blocked (1 h, room temperature) and incubated with primary antibody for overnight at 4°C followed by incubation (2 h, room temperature) with Alexa Fluor secondary antibody. DAPI was used to stain DNA. Images were obtained using fluorescent microscope (Nikon, Japan).

### Protein Extraction and Western Blotting

Protein was isolated from testes and cell specimens using RIPA buffer (Roche, Switzerland) supplemented with a protease inhibitor tablet at the final concentration at 5 mg/ml. Then the sample lysates were centrifuged at 13,000 × *g* for 10 min in 4°C, and supernatant was carefully removed for quantitation assays. The BCA protein assay kit (Pierce Biotechnology, Indianapolis, IN, United States) was used for protein concentration measurement according to the manufacturer’s instructions. Protein samples were then boiled in a sample buffer containing 2% SDS for 10 min followed by incubation on ice.

Protein samples (40 μg/lane) were separated using 10% SDS-PAGE gels, and the protein bands were transferred to nitrocellulose membranes (Bio-Rad). The membrane was blocked with 5% non-fat milk followed by an incubation using primary antibodies (Rad51, Cat: sc-53428 and β-actin from Santa Cruz Biotech) for overnight at 4°C. After washing with TBST, the membranes were incubated with the HRP-conjugated IgG antibody (Santa Cruz Biotech, United States) by enhanced chemiluminescence. Finally, the immunoblots were visualized using the BIO-RAD ChemiDoc XRS imaging system.

### RNA Isolation and Real-Time Quantitative PCR (RT-qPCR)

Total RNA was isolated from testes and cell samples using RNAiso Reagent (TaKaRa, Japan) according to the manufacturer’s instructions and stored in -80°C. The concentration of RNA was detected using NanoDrop 1000 spectrophotometer. One milligram of total RNA samples were reversely transcribed into cDNA using the TransScript RT reagent Kit (Takara, Japan) following the manufacturer’s instructions with random and specific primers (miR-10a). The stem-loop RT primers were designed as follow according to the previous report (Chen et al., 2005): 5′-GTCGTATCCAGTGCAGGGTCCGAGGTATTCGCA CTGGATACGACAACTATA-3′. The PCR primers (forward: 5′-CGCGGCATGAGGTAGTAGGT-3′ and reverse: 5′-GTCGTA TCCAGTGCAGGGTCC-3′) designed by sRNAPrimerDB software.^1^ The reverse transcriptase reaction using Stem-loop primers was performed according to the protocol of our previous studies (Xie et al., 2011). *Gapdh* and U6 were used to normalize the level of mRNA and miRNA expression, respectively. All RT-qPCR were performed using a Biorad Real Time PCR system. The results were calculated using 2^–ΔΔct^ methods. All PCR primers used in this study are presented in Supplementary Table S1.

### Luciferase Reporter Assays

The 3’UTR of *Jarid2*, *Tbx5*, *Sohlh2*, and *Rad51* transcript sequences were obtained from NCBI resources. Oligonucleotides corresponding to conserved *Jarid2*, *Tbx5*, *Sohlh2*, and *Rad51* binding sites predicted by TargetScan7.1 software were annealed and cloned into the dual-luciferase miRNA target expression vector (psiCHECK2). The vectors were transfected into HEK293T cell and co-transfection with a scrambled miRNA sequence was used as a negative control. Briefly, transfection reagent (Sigma, Japan) was added to serum free DMEM medium containing miRNA mimics or negative control, after 15 min, the mixture was added to a 6-well plate. Thirty six hours of post-transfection, luciferase activity was measured with a dual-luciferase assay kit (Promega, United States) following manufacturer’s instructions.

### miR-10a Mimics and Inhibitor Transfection

The miR-10a mimic and inhibitor synthesized by Sangon Biotech (Shanghai, China) were used to be overexpressed and knock-downed miR-10a, then they were transfected into HEK293T, NIH3T3, and GC-1 cell lines using EntransterTM-R4000 (Engreen Biosystem, China) according to manufacturer’s instructions.

### Isolation of Testicular Germ Cells

Testicular germ cells (GCs) were isolated from P21 mice according to the protocol previously described (Chang et al., 2011). Briefly, tunica albuginea was removed from testes under the dissection microscope, seminiferous tubules were detached gently and washed with DPBS for three times. The tubules were digested with 1 mg/mL collagenase IV (Invitrogen) for 3 h and washed with DPBS for three times to remove interstitial cell. Then the samples were sequentially digested with 0.25% Trypsin-EDTA (Gibco) for 5 min at 37°C and then the dispersed testicular cells were filtered through a 40-μm mesh to make single cell suspension. The single cell suspension was pelleted by centrifugation at 350 g for 5 min and resuspended in DMEM/F12 supplemented with 5% (v/v) FBS (Gibco). 5 × 10^6^ cells/mL was seeded into dishes and cultured in a 5% CO_2_ incubator at 34°C. To remove peritubular myoid cells (PMCs), the above cell suspension (including Sertoli cells and PMCs) was cultured for 20 min and the non-adhering cells (mainly comprising Sertoli cells and GCs) were transferred into a new dish. To obtain GCs, the weakly adhering cells were seeded into new dish. The cell purity was verified by RT-qPCR using the marker gene of different type of germ cell and Sertoli cells (Supplementary Figure S1A).

### Statistical Analyses

The experiments were performed at least three repeats and statistical analyses were executed with SPSS software (IBM software). *P <* 0.05 and *P <* 0.01 were used to define the statistical significance of differences between more than two groups. Statistically significant values for *P* < 0.05 and *P* < 0.01 are indicated by single and double asterisk, respectively.

## Results

### Expression Profile of miR-10 Family During the Postnatal Testis Development in Mice

**FIGURE 1:**
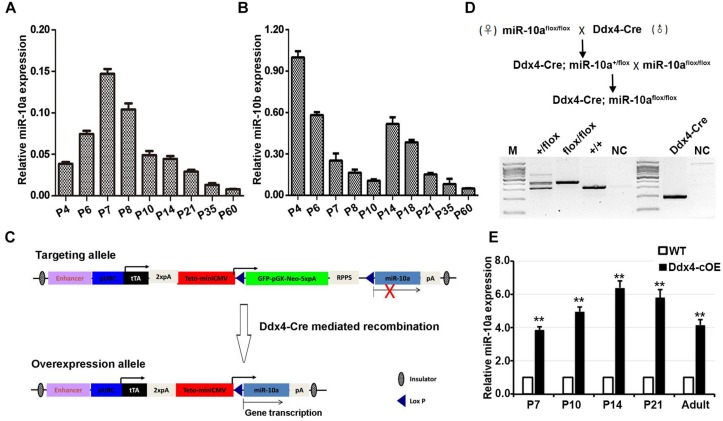
miR-10 expression in developing testes and generation of germ cells miR-10a conditional overexpression transgenic mice. **(A,B)** RT-qPCR analyses of the expression levels of miR-10a **(A)** and miR-10b **(B)** in different ages of mouse testes. The miR-10a/b expression is relative to *U6* and data are presented as mean ± SEM (*n* = 3). **(C)** Strategy for generating a miR-10a overexpressed construct in mice. In the targeting allele, a modified vector CMV-LoxP-GFP-STOP-LoxP containing a synthetic rTA-polyA-TetO-CMV element was insert into upstream of pri-miR-10a, and Cre-mediated recombination leads to the deletion of STOP cassette, which causes pri-miR-10a transcript into mature miR-10a. **(D)** Breeding scheme for germ cells specific miR-10a overexpression mouse model by *Ddx4-Cre* mediation and representative PCR based genotyping results. **(E)** RT-qPCR analysis showing the miR-10a expression level at P7, P10, P14, P21 and adult testes from miR-10a conditional overexpressed (Ddx4-cOE) and WT mice. Data are presented as mean ± SEM (*n* = 3), ^∗∗^*P* < 0.01.

During postnatal testicular development in mice, both undifferentiated and differentiating spermatogonia represent the major male germ cell types at postnatal day 7 (P7), whereas spermatogonia, spermatocytes, and spermatids, are present at adult (P60). To define the miR-10a expression profile in mouse developing testes, we performed RT-qPCR to determine the miR-10a expression levels at P4, P6, P7, P8, P10, P14, P21, P35, and P60 mouse testes. We found that the highest miR-10a expression levels were detected at P7 testes, and displayed a gradually declining expression pattern from postnatal 7 (P7) to adult testes (P60; Figure 1A). This dynamic expression pattern may attribute to spermatogonial miR-10a expression is that the huge increase in total RNA at P7 due to increased germ cell numbers dilutes a somatic signal. To further define the cell specificity of miR-10a expression in the testes, we measured the miR-10a expression levels in isolated germ cells and Sertoli cells by RT-qPCR. Our data showed that miR-10a was higher expressed in different types of germ cells and lower expressed in Sertoli cells (Supplementary Figure S1B), suggesting that miR-10a may have preferential functions in germ cell development. Interestingly, it appears that during the first meiotic entry in the first wave of spermatogenesis (P7 to P14), the expression of miR10a is different from that of miR10b whereas the expression of both miR-10a and miR-10b decreased from P35 to adults (Figure 1B), suggesting a similar expression pattern in steady-state spermatogenesis. Together, our data show that miR-10 family (miR-10a and miR-10b) displayed a distinct dynamic expression patterns during postnatal testis development, and imply the potential distinctive roles of miR-10a and miR-10b may be required for the first wave of spermatogenesis only.

### Generation of miR-10a Germ-Cell Specific Overexpressed Transgenic Mouse Models

To investigate the physiological role of miR-10a in male germ-cell development and spermatogenesis, we generated miR-10a germ-cell specific overexpressed mouse model using Cre/Loxp system. We engineered a targeting construct (Figure 1C) in which containing GFP-STOP cassette flanking with two loxp sites at *Rosa26* locus. pri-miR-10a gene transcript by *Cre* mediated recombination would be activated and lead to miR-10a overexpressed in germ cells. To obtain miR-10a germ cell specific overexpressed mice, we crossed the *miR-10a*-*loxp* transgenic mouse line with *Ddx4-Cre* mouse line, which expresses CRE recombinase in primordial germ cells/prospermatogonia at embryonic day 15.5 (E15.5; Gallardo et al., 2007), to obtain Ddx4-cOE mouse lines. PCR-based genotyping could readily detect the *loxp* allele and genotypes of conditional overexpressed miR-10a mice could be determined in conjunction with PCR-based genotyping on the transgenic *Ddx4-Cre* allele (Figure 1D). To prove that miR-10a overexpressed in male germ cells, we performed RT-qPCR to validate the miR-10a expression levels at P7, P10, P14, P21, and adult testes. As expected, the miR-10a expression levels in Ddx4-cOE developmental testes were significantly higher (∼3–6 folds) than that of in WT testes (Figure 1E), indicating that miR-10a was indeed overexpressed in Ddx4-cOE mouse testes. Moreover, the miR-10a expression levels were also displayed significant increase in selected and unselected testicular germ cells from Ddx4-cOE mice compared to those of WT mice (Supplementary Figure S1C). Together, these data suggest that we successfully generated a transgenic mouse line with miR-10a overexpressed in testicular germ cells.

### Overexpressed miR-10a Results in Male Sterility and Meiotic Defects

**FIGURE 2:**
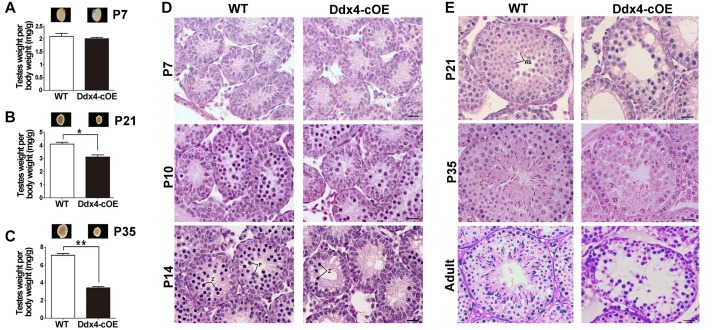
Spermatogenesis disruption and meiotic arrest in germ cell miR-10a overexpressed (Ddx4-cOE) transgenic mice. **(A–C)** Representative testis whole mount images and the ratio of testis weight to body weight (mg/g) at postnatal day 7 (P7), P21 and P35 between WT and Ddx4-cOE mice. Data are presented as mean ± SEM (*n* = 3), ^*^P < 0.05 and ^∗∗^*P* < 0.01. **(D,E)** Representative images showing H&E staining of testes from WT and Ddx4-cOE mice at various developmental time points (P7, P10, P14, P21, P35, and adult). Note that the morphology and total number of germ cells are indistinguishable between WT and Ddx4-cOE testes before P10. Z, zygotene spermatocytes; P, pachytene spermatocytes; RS, round spermatids. Scale bar = 20 μm.

To test whether miR-10a overexpressed in testes could affect male fecundity, we carried out fertility test experiments by mating Ddx4-cOE adult males with fertility-proven WT adult females. During an 8-month breeding, no pups were produced, whereas the control breeding pairs yielded an average of ∼7 pups per litter, suggesting that Ddx4-cOE males are completely infertile. Despite a similar body weight between Ddx4-cOE mice and their littermate controls, testis weights in Ddx4-cOE males were significantly decreased compared to those of age-matched controls from P21 (Figures 2A–C). The ratio of testis/body weight of Ddx4-cOE mice was only ∼50% of that of WT controls at P35 (Figure 2C). Consistently, histological examination revealed that discernable histological differences between Ddx4-cOE and WT testes were observed at as early as P10 testes (Figures 2D,E). In contrast to WT seminiferous tubules that were full of spermatogenic cells at different age of testes (Left panels of Figures 2D,E), histologic examination of testes from Ddx4-cOE males revealed that post-meiotic germ cells (round spermatids, elongating spermatids, and spermatozoa) were completely absent, which demonstrated that spermatogenesis is blocked at meiosis in Ddx4-cOE mice (Right panels of Figures 2D,E). Consistent with testicular seminiferous histologic analyses, no spermatozoa were retrieved from the epididymis of Ddx4-cOE adult mice (data not shown). TUNEL analysis further revealed dramatically increased apoptosis in Ddx4-cOE testis tubules at P10 testes compared to those of WT controls (Supplementary Figure S2). Collectively, these data imply that conditional overexpressed miR-10a in male germ cells cause severe testicular atrophy and male sterility in adulthood by detriment of spermatogenesis.

### Effect of miR-10a Overexpression on Spermatogonial Differentiation and Meiosis Initiation

**FIGURE 3:**
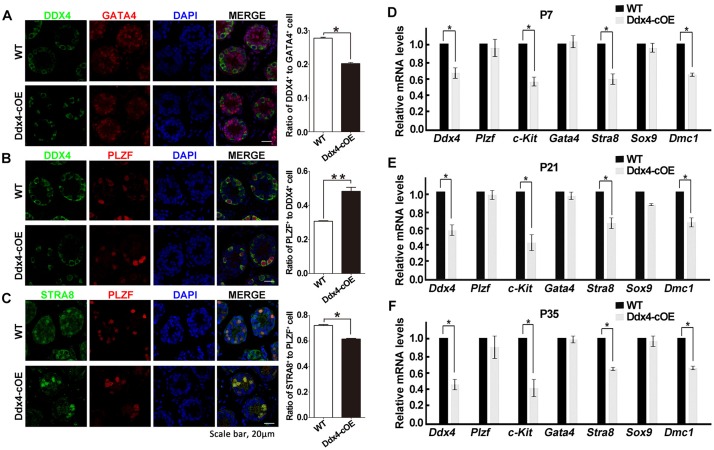
Overexpression of miR-10a affects spermatogonial differentiation. **(A)** Immunofluorescence staining and quantification of DDX4 and GATA4 with DAPI at P7 testes in WT and Ddx4-cOE mice. **(B)** Immunofluorescence staining and quantification of DDX4 and PLZF with DAPI at P7 testes in WT and Ddx4-cOE mice. **(C)** Immunofluorescence staining and quantification of STRA8 and PLZF with DAPI at P7 testes in WT and Ddx4-cOE mice. **(D–F)** Quantitative RT-PCR analysis of seven marker genes mRNA levels in WT and Ddx4-cOE mouse testes at P7 **(D)**, P21 **(E)**, and P35 **(F)**. Data are presented as mean ± SEM (*n* = 3), ^*^P < 0.05 and ^∗∗^*P* < 0.01. All scale bar = 20 μm.

As testicular atrophy could be caused by the compromised function of spermatogonial stem cells (SSCs), we next want to determine whether the spermatogonial differentiation was affected when miR-10a overexpressed in spermatogonia. By immunolabeled testes at P7 with DDX4 (a germ cell marker), PLZF (a marker for undifferentiated spermatogonia that is essential for the maintenance of spermatogonial stem cells), GATA4 (a Sertoli cell marker) and STRA8 (a marker for differentiated spermatogonia and essential for meiosis initiation), we found that the numbers of DDX4-positive cells per Sertoli cells (GATA4 positive cells) were significantly decreased in sections of Ddx4-cOE testes compared with WT controls (Figure 3A). Interestingly, although the expression of PLZF was no significant difference in Ddx4-cOE testis sections compared to those WT controls, the ratio of undifferentiated SSCs (PLZF positive cells) to germ cells (DDX4 positive cells) was significantly increased (*P* < 0.01; Figure 3B), which suggesting the undifferentiated SSCs in Ddx4-cOE testes are prone to differentiate into spermatogonial progenitor that expands and further differentiates than WT testes. However, the ratio of STRA8^+^ cells to PLZF^+^ cells was significantly decreased in Ddx4-cOE testis sections compared to those WT controls (*P* < 0.05; Figure 3C), indicating the less spermatogonial progenitors enter into meiotic process in Ddx4-cOE testes.

Furthermore, we also examined the transcription levels of the several marker genes (germ cell marker, Sertoli cell markers, undifferentiated, and differentiated spermatogonial markers) in Ddx4-cOE and WT developmental testes by quantitative PCR. Consistent with immunostaining results, the expression of *Ddx4*, *c-kit* (a maker for differentiated spermatogonia), *Stra8* (meiosis initiation marker gene) and *Dmc1* (a meiosis recombination regulated gene) were all down-regulated in Ddx4-cOE testes compared to those WT controls at P7, P21, and P35, respectively (Figures 3D–F). As expected, the expression of *Plzf* (undifferentiated spermatogonia marker), *Gata4* and *Sox9* (both are Sertoli cell markers) levels did not display any significant differences between Ddx4-cOE and WT testes at P7, P21 and P35, respectively (Figures 3D–F). Together, these results indicate that miR-10a overexpressed in germ cells may affect the differentiation of SSCs and initiation of the meiosis process.

### miR-10a Is Associated With DSB Repair During Meiotic Process

**FIGURE 4:**
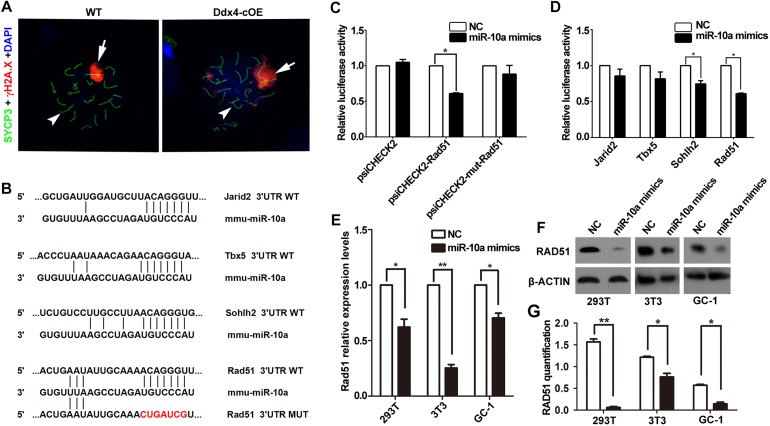
miR-10a targets *Rad51* is essential for DSB repair during meiosis. **(A)** Immunofluorescence staining of SYCP3 and γ-H2AX with DAPI at pachytene spermatocyte spreads in WT and Ddx4-cOE mice. Scale bars = 20 μm. Arrows indicate XY chromosome and arrowheads indicate autosomes. **(B)** A schematic representation of predicted miR-10a binding sites in the mouse *Jarid2*-3’UTR, *Tbx5*-3’UTR, *Sohlh2*-3’UTR and *Rad51*-3’UTR, respectively. **(C)** The relative luciferase activity was assayed following co-transfection of miR-10a mimics with the constructs encoding the wild-type or mutant miR-10a binding site of *Rad51* 3’-UTR into the HEK293T cells. The constructed reporter vectors (psiCHECK2 represent empty vector, psiCHECK2-Rad51 represent Rad51-3’UTR reporter plasmid and psiCHECK2-mut-Rad51 represent Rad51-mut-3’UTR reporter plasmid) are shown on the *x*-axis, and the *y*-axis represents the normalized luciferase activity (firefly luciferase activities were normalized to Renilla luciferase activities). The experiments were performed independently in triplicate. ^*^P < 0.05. **(D)** The expression of luciferase *Jarid2*-3’UTR, *Tbx5*-3’UTR, *Sohlh2*-3’UTR and *Rad51*-3’UTR in HEK293T cells, respectively. The experiments were performed independently in triplicate. ^*^P < 0.05. **(E)** RT-qPCR analyses of *Rad51* expression level in miR-10a mimics co-transfected HEK293T, NIH3T3 and GC-1 cell lines, respectively. Data are presented as mean ± SEM (*n* = 3). ^*^P < 0.05, ^∗∗^*P* < 0.01. **(F)** Protein level of RAD51 in miR-10a mimics co-transfected 293T, 3T3 and GC-1 cell lines by Western blot and protein quantification analyses, respectively. β-ACTIN as loading control. Protein quantification data are presented as mean ± SEM (*n* = 3). ^*^P < 0.05, ^∗∗^*P* < 0.01. NC means negative control.

Due to absence of post-meiotic cells in Ddx4-cOE testes, we next ask whether meiotic processes are affected when miR-10a overexpressed in germ cells. Synapsis of homologous chromosomes is a critical process that is initiated at the zygotene stage and is completed at the onset of the pachytene stage. In males, synapsis defects often lead to meiotic arrest at the pachytene stage. To characterize the cause of meiotic arrest, we analyzed the assembly of synaptonemal complex (SC) by immunolabelling spermatocytes with SYCP3 and SYCP1, which are lateral and central elements of the SC (Pelttari et al., 2001; de Vries et al., 2005). Surprisingly, the expression and localization of SYCP3 and SYCP1 did not display any significant differences in pachytene spermatocytes between WT and Ddx4-cOE testes (Supplementary Figures S3A,B), indicating synapsis of chromosomes is not affected in Ddx4-cOE testes. Thus, we next determined whether double-strand breaks (DSBs) were efficiently processed and repaired. Due to the DNA DSBs can be visualized by γ-H2AX staining at DSB sites during meiotic process, we co-stained γ-H2AX and SYCP3 by chromosome spread assay. Normally, γ-H2AX emerges and globally distributes in the leptotene spermatocytes, persists into zygotene spermatocytes, and is only restricted to the XY body at the pachytene spermatocytes. Similar to that in WT controls, chromosome spread immunofluorescence results showed that global γ-H2AX signals were observed in Ddx4-cOE spermatocytes at the leptotene and zygotene stages (Supplementary Figures S3C,D), which suggests that the formation of DSBs were not impaired. However, the γ-H2AX positive signals were found at the autosomes in Ddx4-cOE pachytete spermatocytes, which should only express at XY chromosome in WT pachynema (Figure 4A and Supplementary Figures S3C,D). Taken together, these results suggest that the chromosome of germ cells could undergo normal synapsis in meiosis, while the pachytene block of germ cells may be associated with the decrease of the repairing capacity of DNA damage in miR-10a overexpressed mouse testes.

### miR-10a Regulates Spermatogenesis in Both Human and Mice by Directly Targeting *Rad51*

**FIGURE 5:**
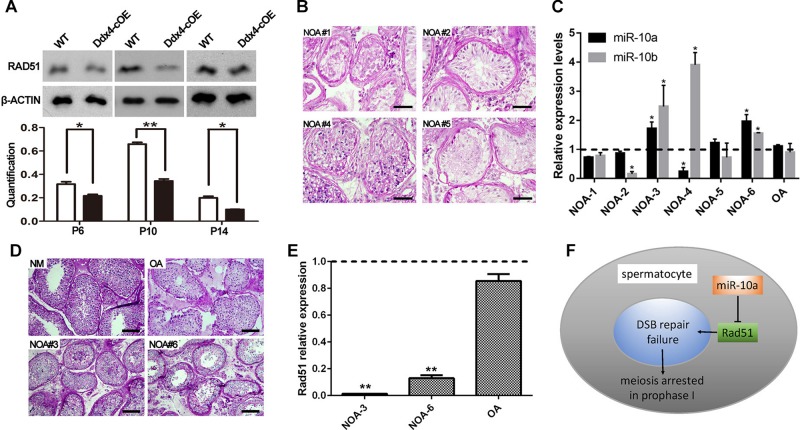
Rad51 as a key target of miR-10a *in vivo* is essential for male fertility in both mouse and human. **(A)** Protein level of RAD51 in WT and Ddx4-cOE testes at P6, P10, and P14 by Western blot and protein quantification analyses, respectively. Protein quantification data are presented as mean ± SEM (*n* = 3). β-ACTIN as loading control. ^*^P < 0.05, ^∗∗^*P* < 0.01. **(B–D)** Histology and hsa-miR-10a/b expression level analyses of testis biopsy tissues from six non-obstructive azoospermia (NOA) patients (NOA#1-6) and one OA patient (as control) by PAS-staining and RT-qPCR. Note that NOA patient #1, 2, and 5 are all showing Sertoli cell only (SCO) phenotype **(B)**, NOA patient #3 and 6 showing post-meiotic arrested phenotype **(D)**. Both hsa-miR-10a and hsa-miR-10b expression levels were showing up-regulated in patient #3 and 6 **(C)**. The experiments were performed technically triplicates. ^*^P < 0.05. Scale bar = 50 μm. NM means normal testis sections from fertile men. **(E)** RT-qPCR analysis of *Rad51* mRNA levels in NOA patients #3, #6, OA patients, respectively. The experiments were performed technically triplicates. ^∗∗^*P* < 0.01. **(F)** A schematic model showing miR-10a regulation of meiotic process in spermatocytes by direct targeting *Rad51*.

Since miRNAs mainly work through regulating the expression of its targeted genes (Carrington and Ambros, 2003). To explore the molecular mechanism of miR-10a in regulation of spermatogenesis, we predicted the potential target genes and the binding sites of miR-10a using three analytical methods such as miRanda, PicTar and TargetScan. Around 300 genes were predicted as the potential targets of miR-10 family (Supplementary Table S2) and GO term analyses showed that those predicted genes are involved in regulation of transcription DNA-templated, male gonad development, etc (Supplementary Figure S4). To validate the prediction results, we selected four candidate genes (*Jarid2, Tbx5, Sohlh2*, and *Rad51*) to perform sequencing alignment and luciferase assays. Sequence alignment displayed the 3′-UTR of *Jarid2, Tbx5, Sohlh2*, and *Rad51* has several consecutive bases complementary to mmu-miR-10a (Figure 4B). Dual luciferase assays revealed that luciferase activity of *Sohlh2* and *Rad51* were significantly decreased in HEK293T cells transfected with miR-10a mimics compared to that of the negative controls (*P* < 0.05), whereas the luciferase activity of both *Jarid2* and *Tbx5* were not significantly changed (Figures 4C,D), suggesting both *Sohlh2* and *Rad51* are indeed targeted by miR-10a in cell lines. Due to the function of *Rad51* was well documented in spermatogenesis and meiotic prophase I (Petukhova et al., 2005; Dray et al., 2011; Dai et al., 2017), we thus selected *Rad51* as a miR-10a targeted gene to further verify the function of miR-10a among different miR-10a mimics transfected cell lines *in vitro*. As expected, qRT-PCR and Western blot revealed that both mRNA and protein levels of *Rad51* are significantly knock-downed in the miR-10a mimics transfected cell lines, including 293T cells, NIH3T3 cells, GC-1 cells, respectively (Figures 4E,F), which further implying *Rad51* gene expression was regulated by miR-10a. To determine the physiological role of miR-10a by regulating *Rad51 in vivo*, we examined the protein levels of RAD51 in testes at P6, P10 and P14, respectively, by Western blot analyses. Consistent with the results of cell lines *in vitro*, the protein levels of RAD51 were indeed significantly decreased in Ddx4-cOE testes compared to those of age-matched WT controls (Figure 5A).

To decipher the function of miR-10a on human spermatogenesis and male fertility in clinic, we next analyzed 6 testicular biopsies from the infertile patients with diagnosis of NOA syndrome by PAS staining and RT-qPCR. Histological analyses revealed that all of NOA patient testes are displayed spermatogenesis defects at some extent, including Sertoli cell only (SCO; NOA#1, 2 and 5), post-meiotic arrested (NOA#3 and 6) and sperm maturation disorder (NOA#4; Figures 5B–D). Strikingly, RT-qPCR further revealed that the expression level of hsa-miR-10a was significantly upregulated in NOA #3 and 6 patient testes (Figure 5C). Notably, the morphology of both NOA#3 and 6 patient testes were displayed meiotic arrest, which are phenocopied the Ddx4-cOE mouse testes. Interestingly, we found that the miR-10b expression was also increased in both NOA#3 and 6 patient testes (Figure 5C), suggesting that miR-10b have similar functions in spermatogenesis and male fertility. These results indicated that, at least in part, miR-10 family is associated with spermatogenesis and meiotic process and responsible for male infertility in human as well. In addition, we also examined the *Rad51* (a targeting gene of miR-10a was identified in mice above) expression levels in NOA #3 and 6 patient testes by RT-qPCR, the expression levels of *Rad51* were very significantly decreased in NOA #3 and 6 testes compared to that of in OA patient testes (Figure 5E). In sum, our data indicate that miR-10a is involved in spermatogenesis and male fertility in both human and mice by directly targeted *Rad51*, overexpressed miR-10a in germ cells may cause DSB repair failure during meiosis and thus lead to male infertility (Figure 5F).

## Discussion

miRNA, a critical regulator of gene post-transcriptional levels, which are involved in multiple developmental processes in many organisms (Hayashi et al., 2008). Several studies have proved that the miRNAs can participate in regulating mammalian spermatogenesis (Yuan et al., 2015b; Niu et al., 2016). For instance, the expression of miR-34/449 cluster was upregulated in the testes upon meiotic initiation in mice, and genetically knockout miR-34/449 clusters resulted in male infertility (Bao et al., 2012; Wu et al., 2014; Yuan et al., 2015b). miR-20 and miR-106a were displayed much higher expression levels in mouse SSCs, compared to differentiating spermatogonia or adult male germ cells (He et al., 2013). Moreover, a high level of miR-34c was found to be presented in adult pachytene spermatocytes and round spermatids (Bouhallier et al., 2010). Therefore, these microRNAs have been proved to exist in testis at different developmental stages and be responsible for spermatogenesis. In this study, we observed that the expression level of miR-10a was the highest at P7 mouse testes when SSCs homed in niches and the differentiation of spermatogonia was initiated (McCarrey, 2013; Figure 1A). It suggested that miR-10a could be participated in the self-renew and differentiation of SSCs. To test this hypothesis, we successfully overexpressed miR-10a in male germ cells, and indeed found that it subsequently affect spermatogonial differentiation and cause DSB repair failure during meiotic process.

Since miRNAs, as small regulatory RNAs, target a wide range of mRNAs, the expression level of those targeted mRNAs are changed when the miRNA knockout or overexpressed *in vivo*. Simultaneous inactivation of two functionally redundant miRNA clusters (miR-34b/c and miR-449) encoding five miRNAs (miR-34b, miR-34c, miR-449a, miR-449b, and miR-449c) led to sexually dimorphic, partial perinatal lethality, growth retardation, and male infertility (Wu et al., 2014). Although the functions of miR-10a are reported based on transient knockdowns and knockout mice by the TALEN system (Stadthagen et al., 2013; Takada et al., 2013), it was not yet reported that the investigation of function in miR-10a using the overexpression animal models. In the present study, we generated, for the first time, a germ cells miR-10a conditional overexpression mouse model by Cre/Loxp recombination system (Ddx4-cOE) to further elucidate the role of miR-10a in spermatogenesis and male fertility. Histologically, the number of germ cells in miR-10a overexpressed testis was significantly reduced compared to the WT controls, and differentiation of germ cells was retarded at pachytene stage in Ddx4-cOE mice. Subsequently, male adult Ddx4-cOE mice suffered from infertility due to germ cells development compromised. It should be noted that genetically loss function of miR-10a did not display observed pathologies (Stadthagen et al., 2013), which may contribute by the function redundancy of other miRNAs that shared same “seed sequence” with miR-10a, like miR-10b. Indeed, miR-10b expression was up-regulated in miR-10a knock down assay, which proved the possibility of redundant function between miR-10a and miR-10b. By contrast, our gain function of miR-10a in germ cells displayed severe infertile phenotype in mice, which demonstrating the critical role of miR-10a from other way during spermatogenesis. Interestingly, we also identified miR-10a was up-regulated in several NOA patient testicular biopsies in clinic and most excitingly those NOA patients phenocopied our miR-10a overexpressed mice, which implying miR-10a plays an essential role in human spermatogenesis.

During spermatogenesis, multiple types of intermediate cells are generated from SSCs. For example, mouse spermatogonia include undifferentiated, differentiating and differentiated spermatogonia, all of which undergo mitotic divisions to amplify cell population as well as step-wise differentiation to prepare for meiosis (Manku and Culty, 2015). miRNAs have been reported to be involved in regulating the process of spermatogonia differentiation processes, such as miR-106b-25, miR-221/222 and miR-202 can increase activity of spermatogonia and reduce the spermatogonia differentiation (Tong et al., 2012; Yang et al., 2013; Chen et al., 2017). Retinoic acid (RA) was identified to promote spermatogonia differentiation and RA can induce miR-10a expression (Koubova et al., 2006; Weiss et al., 2009; Niu et al., 2011). Thus, we inferred that miR-10a is involved in differentiation of male germ cells as well. In fact, in this study, comparing with WT, although the number of PLZF^+^ cell in Ddx4-cOE mouse was not showing obvious differences, the ratio of PLZF^+^ cells to DDX4^+^ cells in Ddx4-cOE mouse P7 testes was significantly higher. These data highly suggested that the number of the differentiated germ cells is distinctly decreased and more undifferentiated SSCs tend to differentiate into spermatogonial progenitor in miR-10a overexpressed in germ cells. Simultaneously, the reduction of number of STRA8^+^ cell in Ddx4-cOE mouse testes showed that the process of meiosis initiation was inhibited. Therefore, our data provide an evidence of single miRNA to regulate SSCs differentiation *in vivo*, which is broaden the horizon of miRNA regulates the differentiation activity of SSCs and the process of meiosis.

A single miRNA can take part in the different biological process by regulating the expression of its target genes (Wei et al., 2013; Wang et al., 2015; Cui et al., 2016). Based on previous studies, miR-10a was reported to be involved in collagen type I generation of hypertrophic scars, embryonic cardiac development, type 2 diabetes mellitus and chronic myeloid leukemia CD34^+^ cells, etc. biological processes by regulating its targeting genes, such as *Pai-1*, *Tbx5*, *Creb1*, *Usf2* (Agirre et al., 2008; Wang et al., 2014; Li et al., 2015; Shan et al., 2016). In present study, we identified around 300 genes were predicted as the candidate targeted genes of miR-10 family by unbiased bioinformatics analyses, and we further chose *Rad51*, a well-documented gene that functioned in spermatogenesis and meiotic process, to validate by dual luciferase reporter assay. Consistently, this *in vitro* result was further confirmed in our *in vivo* study (both mouse and human), in which *Rad51* expression levels were significantly down-regulated in miR-10a overexpressed mouse testes and up-regulated in germ cell arrested patients by Western blot assays. It is worthwhile pointing out that immunohistochemical (IHC) analysis of *Rad51* expression levels in the overexpressed mouse testes would be another layer of evidence for the target of miR-10a, however, unfortunately our RAD51 antibody doesn’t work for IHC experiments and only works for Western blot assays. Nevertheless, given that the fact of *Rad51* plays crucial roles in both mitotic and meiotic recombination, and it can participate in repair of DSBs of DNA in spermatogenesis (Shinohara et al., 1993), we have reasons to believe that miR-10a could be responsible for DNA damage repair during meiosis prophase I by regulating expression of target gene *Rad51* into spermatocytes (Figure 5F).

In summary, we generated germ cells conditional overexpression miR-10a mice by Cre/Loxp recombination system and these mice exhibited male sterility. Our data unequivocally demonstrate an important role for miR-10a in spermatogenesis and meiotic process *in vivo* and provide the knowledge of the function of miR-10a in germ cells development, facilitate investigations into spermatogenesis regulation in both mice and human, and afford a theoretical basis for the treatment of male infertility in clinic. Further studies are required to reveal the precise mechanism of miR-10a highly conserved biological function for spermatogenesis and male fertility.

## Ethics Statement

This study was carried out in accordance with the recommendations of “NIH and European animal care guidelines, Medical Ethics Committee, Tongji Medical College, Huazhong University of Science and Technology” with written informed consent from all subjects. All subjects gave written informed consent in accordance with the Declaration of Helsinki. The protocol was approved by the “Medical ethics committee, Tongji Medical College, Huazhong University of science and Technology.”

## Author Contributions

HG, HW, CC, DD, CY, SX, JZ, and XuH performed the experiments and analyzed the data. XiH and SY reviewed the data and gave extract suggestions on this study. SY and WD initiated the study, designed the experiments, and wrote the manuscript.

## Conflict of Interest Statement

The authors declare that the research was conducted in the absence of any commercial or financial relationships that could be construed as a potential conflict of interest.

## Footnotes

1 http://www.srnaprimer.org

## Supplementary Material

FIGURE S1 The measurement of miR-10a expression levels in different types of testicular cells. **(A)** The purity of isolated cells was verified by RT-qPCR analyses using the marker gene. **(B)** RT-qPCR analyses showing miR-10a relative expression levels in different types of testicular cells. Spg, spermatogonia; Pac, pachytene spermatocytes; RS, round spermatids; ES, elongating spermatids; Ser, Sertoli cells. The miR-10 expression is relative to *U6* and data are presented as mean ± SEM (*n* = 3) **(C)** RT-qPCR analysis showing the miR-10a expression level in unselected testicular cells and selected germ cells from P21 Ddx4-cOE and WT mouse testes. The experiments were performed independently in triplicate. ^∗∗^*P* < 0.01.

FIGURE S2 TUNEL staining analyses of the seminiferous tubules in WT and Ddx4-cOE testes at P10.

FIGURE S3 **(A,B)** Co-immunofluorescent staining for the SYCP1 (red) and the SYCP3 (green) on WT **(A)** and Ddx4-cOE **(B)** different stage of spermatocyte spreads. **(C,D)** Co-immunofluorescent staining for the SYCP3 (green) and the γ-H2AX (red) on WT **(C)** and Ddx4-cOE **(D)** different stage of spermatocyte spreads. Arrows indicate XY chromosome and arrowheads indicate autosomes.

FIGURE S4 The GO term analyses of the predicted target genes in Supplementary Table S2.

TABLE S1 Primer sequences used in this study.

TABLE S2 Prediction of targeted gene list by miR-10 family.
