# Supplementary figures and images for: Overexpression of MicroRNA-10a in Germ Cells Causes Male Infertility by Targeting Rad51 in Mouse and Human
Source: Front Physiol. 2019 Jun 18;10:765. doi: 10.3389/fphys.2019.00765 (PMC6591449; doi:10.3389/fphys.2019.00765)

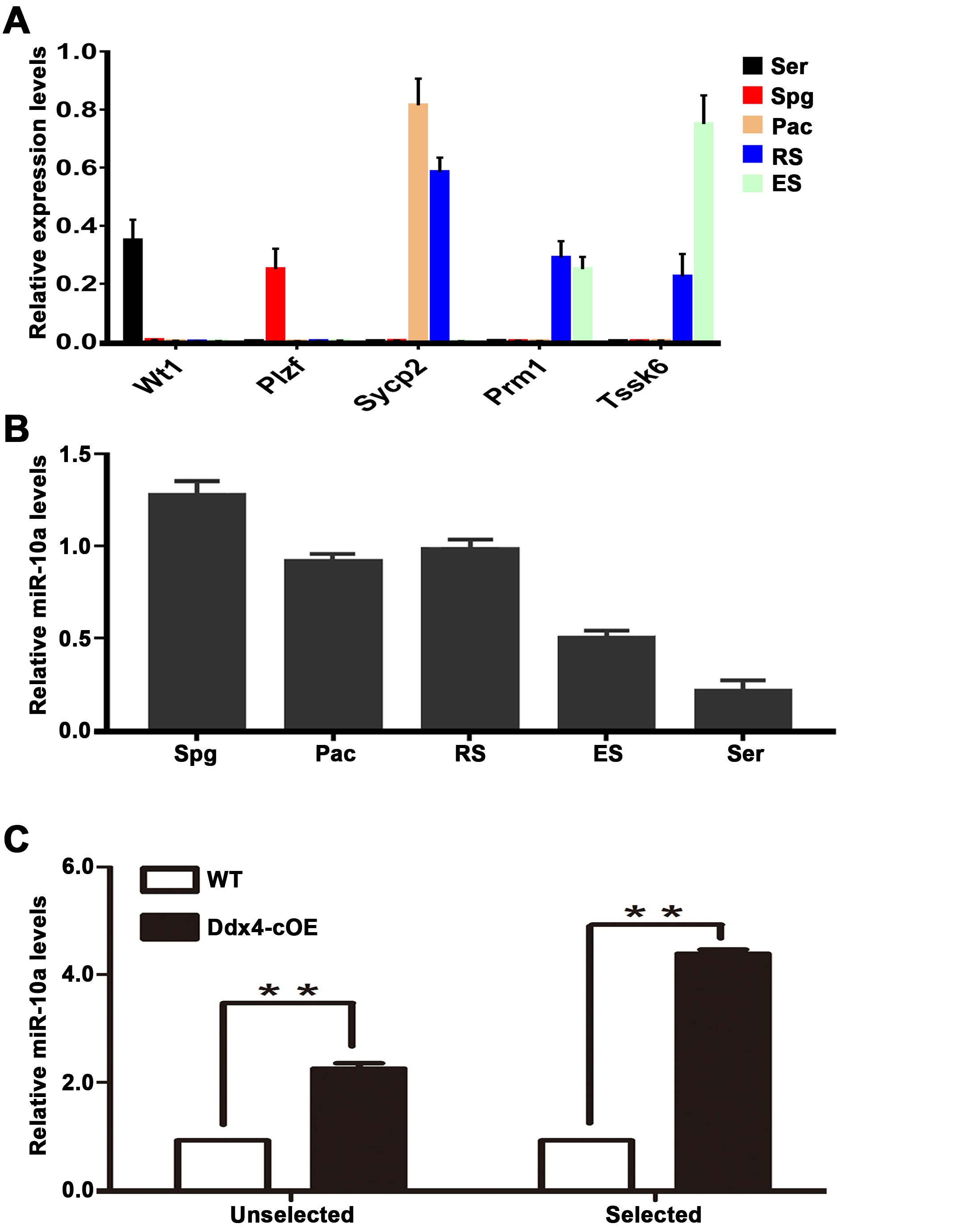

Supplement: FIGURE S1 — The measurement of miR-10a expression levels in different types of testicular cells. (A) The purity of isolated cells was verified by RT-qPCR analyses using the marker gene. (B) RT-qPCR analyses showing miR-10a relative expression levels in different types of testicular cells. Spg, spermatogonia; Pac, pachytene spermatocytes; RS, round spermatids; ES, elongating spermatids; Ser, Sertoli cells. The miR-10 expression is relative to U6 and data are presented as mean ± SEM (n = 3) (C) RT-qPCR analysis showing the miR-10a expression level in unselected testicular cells and selected germ cells from P21 Ddx4-cOE and WT mouse testes. The experiments were performed independently in triplicate. ∗∗P < 0.01. [file Image_1.JPEG]

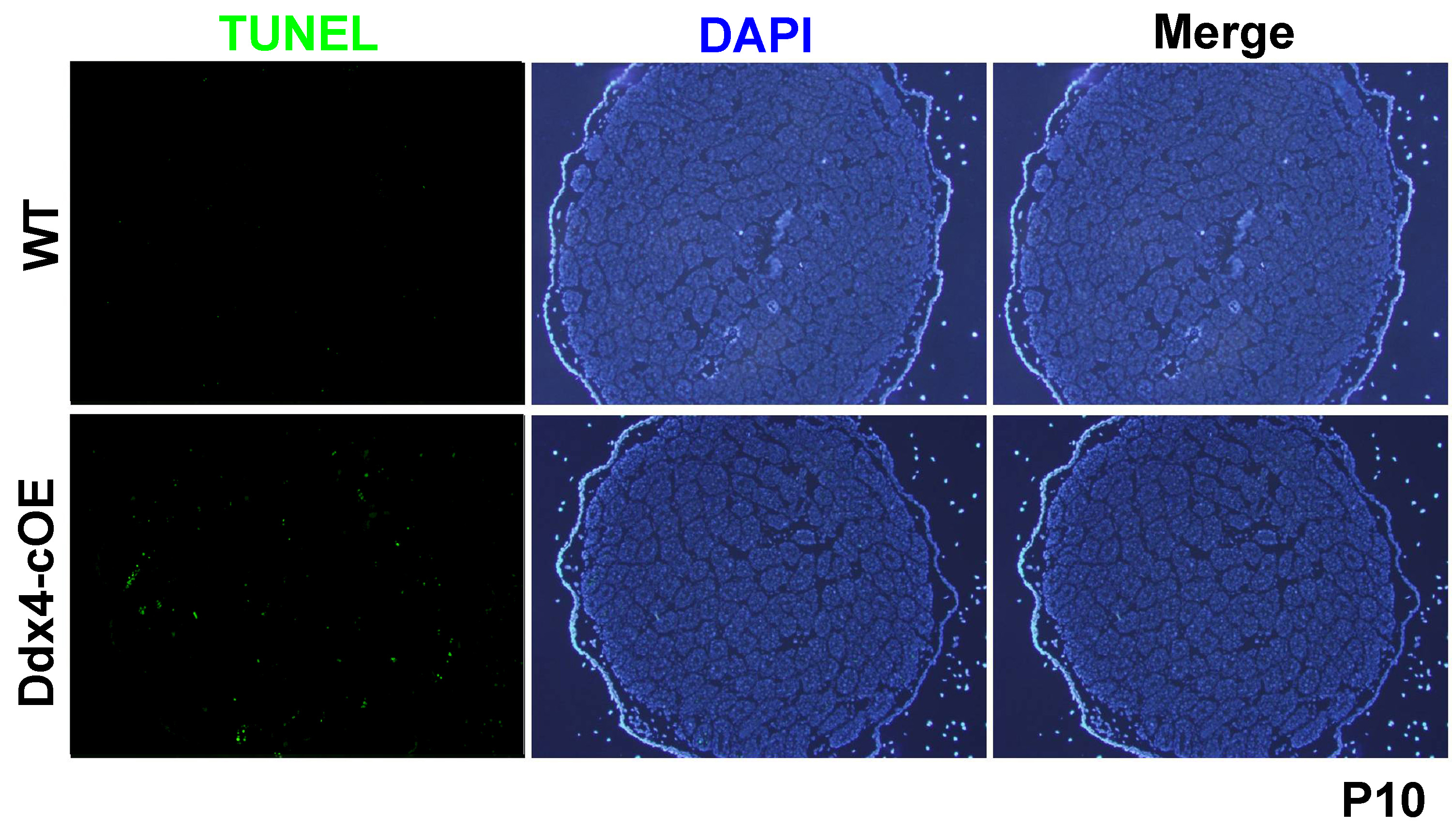

Supplement: FIGURE S2 — TUNEL staining analyses of the seminiferous tubules in WT and Ddx4-cOE testes at P10. [file Image_2.JPEG]

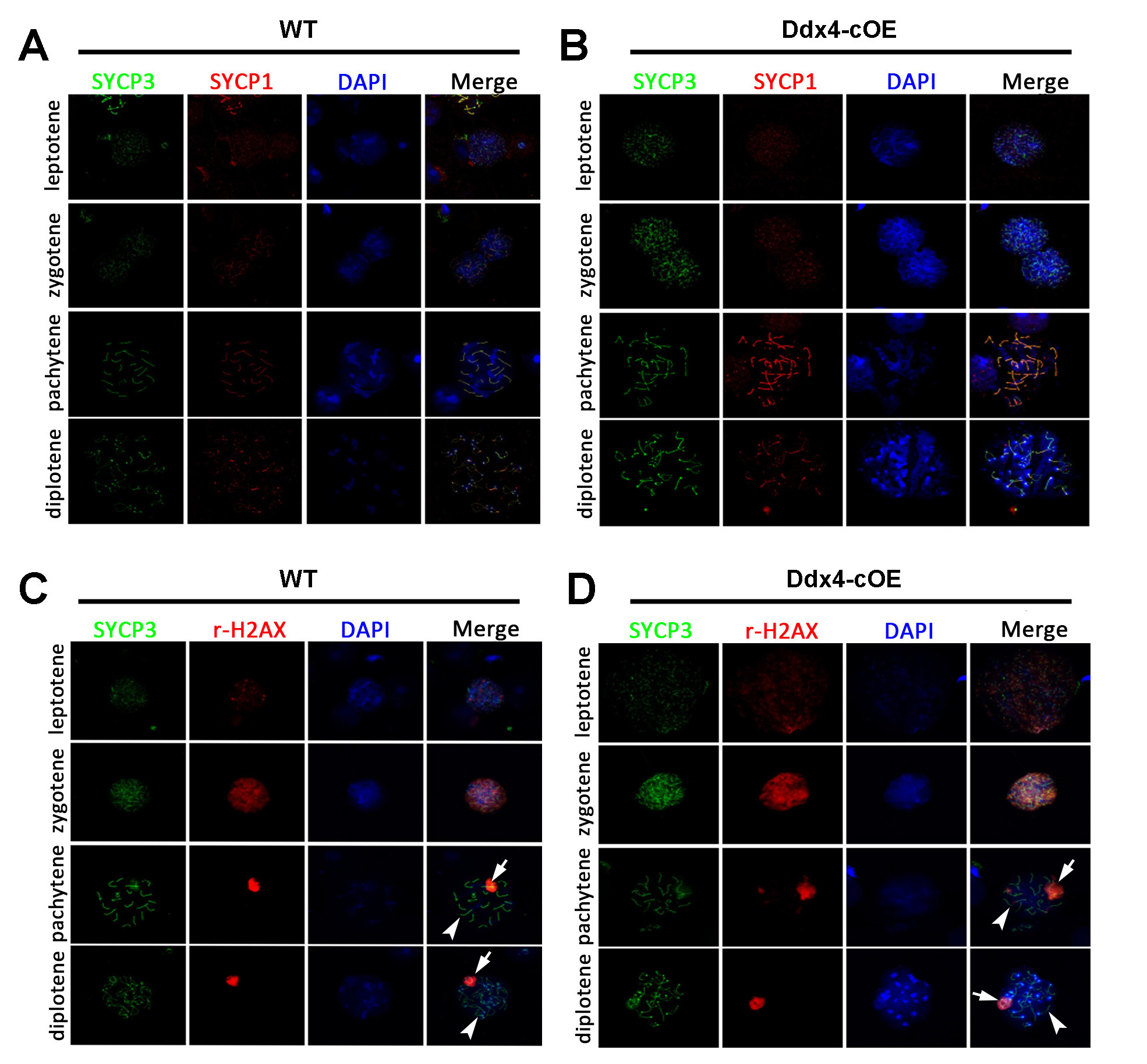

Supplement: FIGURE S3 — (A,B) Co-immunofluorescent staining for the SYCP1 (red) and the SYCP3 (green) on WT (A) and Ddx4-cOE (B) different stage of spermatocyte spreads. (C,D) Co-immunofluorescent staining for the SYCP3 (green) and the γ-H2AX (red) on WT (C) and Ddx4-cOE (D) different stage of spermatocyte spreads. Arrows indicate XY chromosome and arrowheads indicate autosomes. [file Image_3.JPEG]

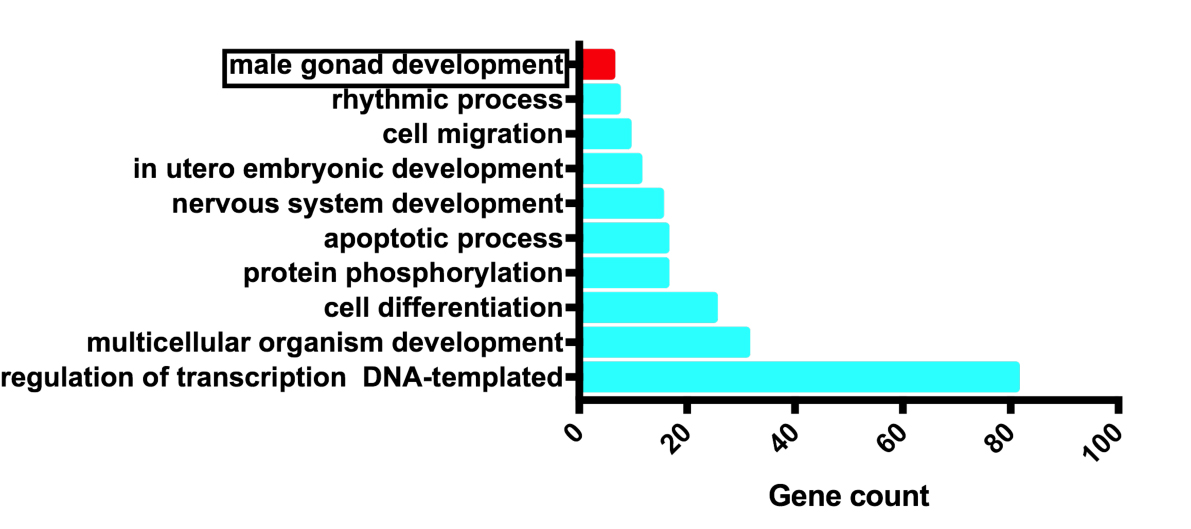

Supplement: FIGURE S4 — The GO term analyses of the predicted target genes in Supplementary Table S2. [file Image_4.JPEG]
