# Supplementary material for: Overexpression of MicroRNA-10a in Germ Cells Causes Male Infertility by Targeting Rad51 in Mouse and Human
Source: Front Physiol. 2019 Jun 18;10:765. doi: 10.3389/fphys.2019.00765 (PMC6591449; doi:10.3389/fphys.2019.00765)
Supplement: TABLE S1 — Primer sequences used in this study. [file Table_1.DOC]

**Table S1. Primer sequences used** in this study.

| **Gene symbol** | **Forward（5’ 3’）** | **Reverse（5’ 3’）** | **Usage** |
| --- | --- | --- | --- |
| Sox9 | GCGGGTCAACGGCTCCA | GCCATTCTTCACCGACTTCC | qPCR |
| Stra8 | CTCCTCCTCCACTCTGTTGC | GCGGCAGAGACAATAGGAAG | qPCR |
| Plzf | CTGGGACTTTGTGCGATGTG | CGGTGGAAGAGGATCTCAAACA | qPCR |
| c-kit | GAATCTCCGAAGAGGCCAGAA | GCTGCAACAGGGGGTAACAT | qPCR |
| Gata4 | CGGAAGCCCAAGAACCTGAATAAAT | GCTGCTGTGCCCATAGTGAGATGAC | qPCR |
| Ddx4 | GTTTGCATCTGTTGACACGAGGA | CAACTGGATTGGGAGCTTGTGA | qPCR |
| Dmc1 | CCCTCTGTGTGACAGCTCAAC | GGTCAGCAATGTCCCGAAG | qPCR |
| Rad51 | CATTGGAGGGAACATCATCG | GGAGTCAGTCTTTGGCATCG | qPCR |
| Gapdh | TGCCACCCAGAAGACCGT | AGGGATGACTTTGCCCACAC | qPCR |
| Ddx4-Cre | CCAGTTTGGTCATTCAGTTCGA | AAAAGGGTTTGGCGTTGTTC | Genotyping |
| miR10a-loxp | GGTGTTCTTGTGCGTGTGATGT | CCTCAGAAGCCATAGAGCCCAC | Genotyping |
| Wt1 | TAGTTAGGACAGAGAGGAGCC | TGGAACTGAGTCCTCCACATC | qPCR |
| Plzf | ATCTGCACGGAATACTGCCC | TGGTTCTAGCTCGCCTTTGC | qPCR |
| Sycp2 | GCAAGAACCTCCCGACTCAA | AGCAGAGCCTTTTCCTCTTTCA | qPCR |
| Prm1 | ATGGCCAGATACCGATGCTG | TTATGGTGTATGAGCGGCGG | qPCR |
| Tssk6 | AAGAGAGAGAAGCATTGCGCC | CCCACGGTTTCTCTGTAGCAT | qPCR |
